# Pressing ahead: developing and testing of new measures in implementation science

**DOI:** 10.1186/1748-5908-10-S1-A14

**Published:** 2015-08-20

**Authors:** Maria E Fernandez, Shuting Liang, Sara R Jacobs, Stephen H Taplin, Bryan J Weiner

**Affiliations:** 1 School of Public Health, University of Texas Health Science Center at Houston, Houston, TX 77030, USA; 2 Department of Behavioral Sciences and Health Education, Rollins School of Public Health, Emory University, Atlanta, GA 30322, USA; 3 Social & Health Organizational Research and Evaluation, RTI International, Atlanta, GA 30341, USA; 4 Healthcare Delivery Research Program, National Cancer Institute, Rockville, MD 20850, USA; 5 Department of Health Policy and Management, Gillings School of Global Public Health, University of North Carolina at Chapel Hill, Chapel Hill, NC 27599, USA

## 

Measurement forms the foundation of any scientific field; yet, systematic reviews reveal that many available measures of implementation context, process, and outcomes lack reliability or validity. An urgent need exists for psychometrically strong measures in implementation science; without them, the field cannot produce cumulative knowledge about implementation barriers, facilitators, processes, or generate sound evidence about which implementation strategies work best, when, and for whom. In this panel session, three researchers reported on their efforts to develop and test new measures of constructs featured in the Consolidated Framework for Implementation Research (CFIR). Maria Fernandez described the work of the CDC/NCI-funded Cancer Prevention and Control Research Network to create measures for seven constructs in the inner-setting domain of CFIR and assess the psychometric properties of those measures using data from a multi-state sample of community health centers. Shuting Liang reported on the Network's effort to develop and assess measures of selected constructs in other CFIR domains and discussed the inter-relationships of these constructs at both the individual and clinic level of analysis. Sara Jacobs explored in two different study contexts the psychometric properties of, and measurement issues associated with, a new theory-based measure of implementation climate. Building on the presentations, Stephen Taplin moderated a discussion between panelists and participants about the role of theory in measurement, the challenges of adapting existing measures, the implications of item-wording choices, the effects of context on measurement properties, and the measurement of organization-level constructs using individual-level data. Participants learned about new measures they could use in their own research; in addition, they engaged in dialogue about needs, opportunities, challenges, and recommended practices in measurement in implementation science.

## Developing measures to assess constructs from the inner setting of the consolidated framework for implementation research

Implementation scientists and practitioners alike need reliable, valid measures of contextual factors that influence implementation success. Yet few existing measures demonstrate reliability or validity. To meet this need, we developed and assessed the psychometric properties of measures of several constructs within the inner setting domain of the Consolidated Framework for Implementation Research (CFIR).

We searched the literature for existing measures for seven **inner-setting domain** constructs (available resources for implementation, culture overall, culture stress, culture effort, implementation climate, learning climate, and readiness for implementation). We adapted items for the healthcare context, pilot-tested the adapted measures in 4 CHCs, and fielded the revised measures in 78 CHCs in seven states (N = 327 respondents). To psychometrically assess our measures, we conducted confirmatory factor analysis (structural validity), assessed inter-item consistency (reliability), computed scale correlations (discriminant validity), and calculated inter-rater reliability and agreement (organization-level construct reliability and validity).

CFAs for each construct exhibited good model fit (CFI>0.90, TLI>0.90, SRMR<0.08, RMSEA<0.08), with factor loadings exceeding .40. A seven-factor CFA failed to converge but a five-factor CFA with the three culture constructs modeled as a single factor exhibited a good model fit when resources and implementation climate were allowed to covary (CFI = 0.848, TLI = 0.835, SRMR = 0.079, RMSEA = 0.065). Scale reliabilities ranged from good (0.7≤α<0.9) to excellent (α≥0.9). Scale correlations fell below .85, indicating discriminant validity. Inter-rater reliability and agreement were sufficiently high to justify measuring constructs at the clinic level.

Our findings provide psychometric evidence in support of the CFIR inner setting measures. Our findings also suggest that the inner setting measures can be aggregated to the clinic level and at the CHC system level. Measurement of inner-setting constructs can be useful in better understanding and predicting implementation in CHCs and can be used to identify targets of strategies to accelerate and enhance implementation efforts in CHCs.

## Measuring constructs from the consolidated framework for implementation research in the context of increasing colorectal cancer screening at community health centers

The Consolidated Framework for Implementation Research (CFIR) is a comprehensive meta-framework widely applied to implementation related studies. Yet, few have used validated measures to operationalize constructs in CFIR in real-life settings. In this study, we operationalized selected CFIR constructs in an assessment to identify factors influencing implementation of evidence-based practices for increasing colorectal cancer screening in Community Health Centers (CHC).

We selected 16 constructs from **all five domains** of CFIR. Measures were developed and tested in a cross-sectional survey with CHCs' clinical staff and leaders respectively. We performed a separate confirmatory factor analysis (CFA) for measures with three or more items, computed inter-item consistency (Cronbach's alpha), inter-rater reliability (ICC) and agreement (r_WG(J)_) statistics, and assessed construct validity via inter-correlations among constructs at individual and organizational levels.

A total of 277 individuals and 59 CHC clinics were included in the analysis. CFA showed satisfactory structural validity (CFI>0.90, TLI>0.90, SRMR<0.08, RMSEA<0.08); all measures showed reasonable reliability (alpha>0.70). The ICCs (>0.1) and r_WG(J)_s (>0.75) suggest it appropriate to aggregate individual responses by computing clinic means. Results also suggest good construct validity at both individual and clinic levels. Inner setting and process-related constructs are correlated with most variables across domains; correlations between outer setting and intervention characteristics and other domains vary more noticeably by construct.

Our study is one of the first to quantitatively measure constructs from all five domains of CFIR and demonstrate their psychometric properties. We depicted their inter-correlations at multiple levels, which set the foundation for establishing predictive models, causal pathways and developing interventions that target these factors. These findings could contribute to further development of the CFIR.

## Measuring implementation climate: context matters

Implementation climate is considered a primary driver of effective innovation implementation. However, issues surrounding the measurement of implementation climate, or the extent to which organizational members perceive that innovation use is expected, supported and rewarded by their organization remain. Specifically, we examined whether implementation climate can be measured as a global construct, whether individual or group-referenced items should be used to measure implementation climate, and whether implementation climate can be assessed at the group or organizational level.

This research includes two cross-sectional studies with data collected via surveys. The first study assessed the climate perceptions of physicians participating in the National Cancer Institute's Community Clinical Oncology Program. The second study assessed the climate perceptions of children's behavioral health clinicians implementing a treatment innovation. To address our first objective, we used confirmatory factor analysis. To address the second and third objectives, we followed an established protocol, which includes exploratory factor analysis and correlations to assess differences between items and intra-class correlations, inter-rater agreement statistics to determine the appropriate level of measurement.

Results indicated that implementation climate can be measured as a global construct reflecting expectations, support and rewards for innovation use. Results were mixed about how implementation climate should be measured and at what level. In our first study, where physicians were geographically dispersed and practice independently, there were no differences based on the type of items used, and little basis for assessing climate at the organizational level. In the second study, in which clinicians practice in a central location and interact more frequently, group-referenced items seemed more appropriate and implementation climate could be assessed at the organizational level. In sum, results were (study) context-specific.

These results advance implementation science by addressing measurement issues regarding a key construct that appears in widely used conceptual frameworks in the field.

